# As-Flux-Induced Diameter Control in GaAs Nanowires

**DOI:** 10.1021/acs.jpcc.5c03887

**Published:** 2025-09-23

**Authors:** Ziyue Yin, Haotian Zeng, Giorgos Boras, Raghavendra R. Juluri, Huiwen Deng, Hui Jia, Chong Chen, Stephen Church, Anton Velychko, Fahad Alghamdi, Jae-Seong Park, Mingchu Tang, David Mowbray, Patrick Parkinson, Ana M. Sanchez, Huiyun Liu

**Affiliations:** † Department of Electronic and Electrical Engineering, 4919 University College London, London WC1E 7JE, U.K.; ‡ Department of Physics, 2707 University of Warwick, Coventry CV4 7AL, U.K.; § Department of Physics and Astronomy and the Photon Science Institute, 5292 The University of Manchester, Manchester M13 9PL, U.K.; ∥ School of Mathematical and Physical Sciences, 7315 The University of Sheffield, Sheffield S3 7RH, U.K.; ⊥ King Abdulaziz City for Science and Technology, Riyadh 11442, Kingdom of Saudi Arabia

## Abstract

Controlling the diameter of self-catalyzed III–V nanowires is important for tailoring their performance in optoelectronic applications. Here, we investigate the impact of abrupt or gradual increase of the V/III flux ratio on the GaAs nanowire diameter. A dynamic model of nanowire diameter is developed to explain the changes induced by flux ratio modulation: (i) shrinkage of the catalyst droplet under elevated As flux and (ii) convergence toward a critical diameter governed by the flux ratio during subsequent nanowire elongation. The different diameter behaviors observed under abrupt or gradual flux increase are elucidated by this relationship, through which we present a quantitative analysis of the relationship between the nanowire diameter and the V/III flux ratio. Epitaxial Ge shells were grown around the modulated-diameter GaAs cores to investigate any impact on the morphology and quality of the group-IV shell. The Ge shell is found to maintain a uniform thickness, regardless of the diameter of the GaAs core. High-resolution annular dark-field scanning transmission electron microscopy reveals Ge shell sidewalls indexed to the {112} planes and rotated by 47° relative to the GaAs core facets, while energy-dispersive X-ray spectroscopy confirms slight Ge interdiffusion into the GaAs core. This work provides a predictive framework for controlling the diameter evolution under varying flux ratios and provides insights into III–V/IV heterointegration.

## Graphical abstract

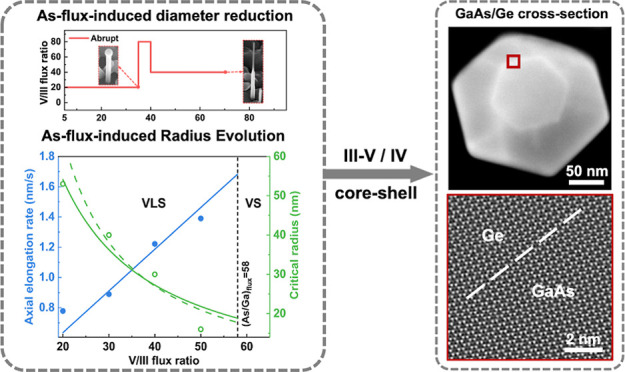

## 1 Introduction

One-dimensional semiconductor nanowires (NWs) exhibit a number of novel and advantageous properties, including a small footprint, a high surface-to-volume ratio, and the ability to accommodate elastic strain relaxation. Due to their large strain tolerance, NWs can be monolithically grown on a variety of substrates,^1–4^ including silicon (Si), rendering them potentially compatible with complementary metal-oxide-semiconductor (CMOS) technology.^5,6^ NWs are thus important building blocks for next-generation functional devices.^7,8^ They also provide flexibility in structural design via the ability to form dislocation-free core–shell heterostructures.^9^ Among the available methods of NW synthesis, the vapor–liquid–solid (VLS) method has attracted great interest, enabling growth of a wide range of semiconductor NWs.^10,11^

Understanding the fundamental petameters of NWs, such as their diameter, is crucial for tailoring their optoelectronic properties and guiding optoelectronic device designs. For example, reducing the diameter of GaAs NWs below 25 nm is known to induce quantum confinement effects, thereby modulating the photon energy of the allowed optical transitions.^12–14^ Modulated-diameter III–V NWs are also important for developing radial core–shell heterostructures, where the core diameter determines the thickness and strain distribution of the surrounding shell layers as well as the overall device structure. For instance, GaAs/AlGaAs core–shell NW lasers require a core diameter exceeding ∼300 nm to achieve the single-mode transverse optical confinement within the GaAs core.^15^ In addition, axial diameter modulation in III–V NWs offers further functionality for optoelectronic applications, including single-photon source, photodetectors, and solar cells.^16–18^ For example, pure single-photon emission has been demonstrated from diameter-modulated GaAs NWs embedding a single InAs quantum dot (QD), yielding high source efficiency and broadband spectral coverage.^19^ Notably, dot-in-wire III–V heterostructures have exhibited localized excitonic emission with QD-like characteristics,^20^ and the brightness of such emission sources can be further enhanced by using diameter-controllable III–V NWs.^16,21^

One effective approach to reducing the diameter of self-catalyzed GaAs NWs is to modulate the incoming elemental fluxes.^22^ For example, the average diameter of self-catalyzed GaAs NWs was shown to decrease from ∼160 nm to ∼50 nm when the gallium (Ga) flux was reduced through halving the Ga growth rate.^23^ Furthermore, ultrathin GaAs NWs with diameters down to 20 nm have been achieved by reducing the Ga droplet size prior to increasing the V/III flux ratio.^13^ This approach involves a two-step growth method to induce an abrupt diameter reduction in individual NWs: first, droplet shrinkage under a step increase in As flux and, second, elongation of a thin NW segment under a high As flux. A small Ga droplet size and an elevated V/III flux ratio during self-catalyzed GaAs NW growth are known to play a critical role in achieving thinner NW diameters.^24–26^ However, the underlying mechanism responsible for the abrupt diameter reduction remains unclear, specifically the combined effect of the droplet shrinkage and the NW elongation under a high flux ratio.

It is important to underline that one of the most interesting NW properties is the ability to form core–shell heterostructures, where a shell is synthesized radially to surround the initially formed core NW.^27^ The role of this shell is 3-fold; it enhances radial quantum confinement by acting as a barrier, it provides passivation for the dense surface states which may result in nonradiative carrier recombination, and it protects the core from oxidation.^28,29^ To date, hybrid group III–V/IV radial heterostructures have been successfully demonstrated using uniform-diameter III–V cores as platforms for the integration of group-IV materials, for example, GaP/Si,^30^ GaAs/Si,^31^ and GaAs/Ge.^32^ However, the influence of the diameter modulation of the III–V core on the integration quality and the resulting group-IV shell morphology remains underexplored.

Here, we report the self-catalyzed growth of GaAs NWs with a diameter reduction on Si(111) substrates and the subsequent development of GaAs/Ge core–shell NWs. During GaAs NW growth, an abrupt increase in the V/III flux ratio from 20 to 80 induces rapid shrinkage of the catalyst droplet, followed by the growth of a thin elongated segment at a reduced flux ratio of 40. This segment exhibits a significantly smaller diameter of 60 nm compared to the initial GaAs stem diameter of 111 nm. In contrast, when the V/III flux ratio is gradually increased from 20 to 80, a tapered morphology with a slight decrease in diameter during each growth interval is observed. The Ga droplet is eventually depleted, resulting in the termination of axial growth and the onset of vapor–solid (VS)-dominated radial expansion. Based on these experimental results, the observed diameter changes in both methods are explained by the combined effects of droplet shrinkage dynamics and NW diameter reduction as a function of the V/III flux ratio. Following GaAs core formation, a Ge shell with a uniform thickness along the NW axis is subsequently grown. Transmission electron microscopy (TEM) reveals a sharp GaAs/Ge core–shell interface and a uniformly thick Ge shell. Notably, the heteroepitaxial Ge shell displays well-defined {112} side facets rotated by 47° relative to the GaAs core. Energy-dispersive X-ray spectroscopy (EDX) confirms a uniform distribution of both the III–V and group-IV elements across the core–shell structure. This work demonstrates the effective diameter reduction of self-catalyzed GaAs NWs, along with the successful heteroepitaxial growth of Ge shells on reduced-diameter GaAs cores.

## 2 Experimental Methods

Self-catalyzed GaAs NWs were grown on p-type Si(111) substrates via the VLS method using a solid-source III–V molecular beam epitaxy (MBE) system. Si(111) substrates underwent a pregrowth treatment to ensure a pristine surface for NW nucleation; the detailed cleaning procedure was described in our previous work.^33^ The GaAs NW growth consists of two stages: (i) a 30 min growth of the GaAs stem NW and (ii) modulation of the V/III flux ratios during the subsequent GaAs NW elongation. Growth was initiated by depositing the Ga catalyst onto the substrate for 5 min under a Ga beam equivalent pressure (BEP) of 8.4 × 10^–8^ Torr, corresponding to a planar growth rate of 0.85 Å/s, at a substrate temperature of 600 °C. Unless otherwise specified, both Ga flux and substrate temperature were kept constant throughout the GaAs NW growth. For the 30 min GaAs stem NW growth, the As flux was introduced into the chamber at a BEP of 1.68 × 10^–6^ Torr, yielding a V/III flux ratio of 20 and a planar growth rate of 3.16 Å/s. Following the formation of the GaAs stem NWs, two approaches were used to modulate the V/III flux ratio, i.e., increasing the As flux while keeping the Ga flux constant, either through an abrupt increase or a gradual increase. In both methods, a final 5 min As flux step was introduced after terminating the Ga supply to ensure complete consumption of the remaining catalyst droplets. In addition, two sets of reference GaAs NWs are grown under stable V/III flux ratios of 20 and 30 for 30 and 60 min, respectively. Subsequently, GaAs NWs exhibiting pronounced diameter reduction were transferred to a group-IV solid-source MBE for VS growth of the Ge shell. The group-IV MBE was connected to the III–V MBE via an ultrahigh vacuum tunnel in a joint system. The Ge shell was grown for 90 min at a substrate temperature of 400 °C under a Ge BEP of 2.5 × 10^–8^ Torr, corresponding to a planar growth rate of 0.40 Å/s. This growth temperature was chosen to preserve the structural integrity of the GaAs core and to promote a smoother Ge shell morphology. As demonstrated in our previous study, Ge shell growth at this temperature window leads to smoother side facets and minimizes defect formation.^32^

A preliminary assessment of the morphology of the GaAs NWs and GaAs/Ge core–shell NWs was performed by using a Zeiss Cross Beam XB 1540 SEM operating at 20 kV, followed by TEM analysis, where the GaAs/Ge core–shell NWs were directly transferred to a lacey carbon grid. Ultramicrotome sectioning was used to produce an 80 nm thick slice cut from a resin-embedded NW. All samples were examined using a JEOL-2100 and a doubly corrected JEOL-ARM microscope, both operating at 200 kV. EDX analysis was performed to determine the elemental composition. X-ray diffraction (XRD) and Raman analysis were conducted to obtain information about lattice properties. XRD was performed using a PANalytical X’Pert^3^ MRD facility in a 2Theta-Omega scanning mode. Raman analysis was conducted with a Renishaw inVia Qontor confocal Raman microscope at 300 K, under an excitation wavelength of 532 nm. Each Raman measurement was accumulated 10 times. In addition, room-temperature photoluminescence (PL) measurements were conducted by using a Nanometrics RPM2000 system with a 532 nm laser source.

## 3 Results and Discussion

The changes in the V/III flux ratio during GaAs NW growth using an abrupt or gradual increase are summarized in Table 1. Statistical analysis of these samples was performed based on SEM images in Figure S1 of the Supporting Information, with the results presented in Figure S2. Two representative samples, A1 and B1, grown by abrupt and gradual methods, are selected to illustrate the resulting NW morphologies. The evolution of NW morphology in relation to the V/III flux ratio is summarized in Figure 1a and b, with the corresponding flux ratio profiles presented in Figure 1c.

**Table 1:** V/III Flux Ratio Profiles for GaAs Nanowire Growth

sample	30 min stem V/III ratio	catalyst droplet shrinkage ratio (time)	thin GaAs NW elongation ratio (time)
A1	20	80 (5 min)	40 (30 min)
A2	20	120 (5 min)	40 (10 min)
A3	20	80 (7.5 min)	40 (10 min)
A4	20	80 (5 min)	50 (10 min)
B1	20	20–80 (2 min per 10 increment)	20–80 (8 min per 10 increment)
C1	20	constant ratio (30 min)	reference samples
C2	30	constant ratio (60 min)	

**Figure 1:**
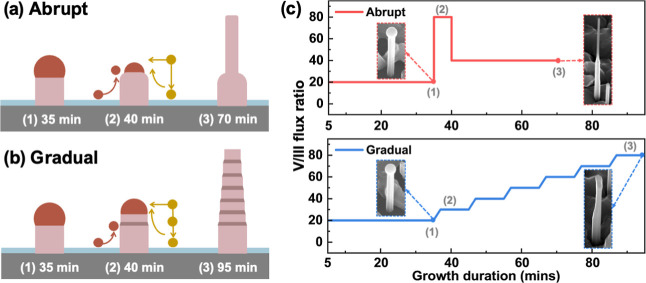
Schematics illustrating the two methods for increasing the V/III flux ratio during GaAs nanowire growth. (a,b) Growth processes for GaAs nanowires under (a) an abrupt and (b) a gradual increase in As flux (Ga flux is kept constant), showing the nanowire evolution over time. In panel (b), the brown segment denotes the region grown under a gradually increasing V/III flux ratio, with brown and yellow arrows representing the Ga and As fluxes, respectively. (c) The evolution of the V/III flux ratio as a function of time. Representative SEM images of GaAs nanowires at the final stage of the growth as shown in (a) and (b) are also included in (c).

For the growth of sample A1 using the abrupt method, a significant diameter reduction was achieved by (i) reducing the Ga droplet volume through an abrupt increase in the V/III flux ratio from 20 to 80 and (ii) continuing NW elongation at a reduced V/III ratio of 40. Due to the negligible solubility of As in liquid Ga, the Ga droplet at the NW apex can be considered to consist solely of Ga and serves as a Ga reservoir.^34^ The sudden increase in As flux consumes the Ga content, leading to a significant volume reduction during the 5 min period of elevated As beam flux, as illustrated in step (2) of Figure 1a. The reduced droplet size achieved by this step can dynamically either inflate or shrink further, depending on the NW radial dimension determined by the subsequent V/III flux ratio. Preshrinking the droplet promotes a more rapid diameter reduction and helps prevent the formation of a tapered morphology.

In the second growth step of sample A1, the V/III flux ratio was reduced to 40, and NW growth continued for an additional 30 min. As shown in Figure 1c, a pronounced diameter reduction was observed at the upper section of the NW, confirming the abrupt diameter reduction achieved through the change in As flux. The diameter variation within individual GaAs NWs is induced by dynamic changes in the Ga droplet size during VLS growth. As reported in previous study, the droplet size is determined by a combination of direct Ga flux impingement from the vapor phase and adatom diffusion from the NW sidewalls.^25^ Following an abrupt increase in As flux, the VLS growth regime becomes increasingly dependent on the sidewall diffusion of Ga adatoms. To elucidate the resulting diameter changes, we applied equations proposed by Dubrovskii et al., which provide a predictive description of how growth parameters affect the NW diameter, as illustrated in Figure 2a.^24,35,36^ Assuming the droplet size varies with time and the contact angle β remains independent of the NW radius, the evolution rate d*R*/d*t* of NW radius *R* can be expressed by eq 1.

1 $$\frac{dR}{dt}=\frac{\Omega_{Ga}}{\Omega_{GaAs}f\left(\beta \right)}\left[\left(\chi_{Ga}\nu_{Ga}-\frac{dL}{dt}\right)+\frac{{\left(\frac{\lambda }{R}\right)}^{p}\left(2\nu_{Ga}\sin\alpha \right)}{\pi }\right]$$

Here, Ω_Ga_ denotes the elementary volume of Ga in the liquid phase, while Ω_GaAs_ represents the volume per GaAs pair in the solid phase. The term *f*(β) is a geometric factor associated with contact angle β. χ_Ga_ describes the geometrical function of the droplet, and ν_Ga_ is the Ga deposition rate in nm/s. λ refers to the characteristic diffusion length of Ga atoms, either along the NW sidewall or on the substrate surface. The exponent *p* depends on the diffusion mechanism; for example, *p* = 1 corresponds to adatom diffusion along the NW sidewall, whereas *p* = 2 refers to diffusion from the substrate surface. The term d*L*/d*t* represents the NW axial elongation rate, which is limited by the impingement and desorption of As species from the Ga droplet. These parameters are illustrated in the schematic model shown in Figure 2a.

**Figure 2:**
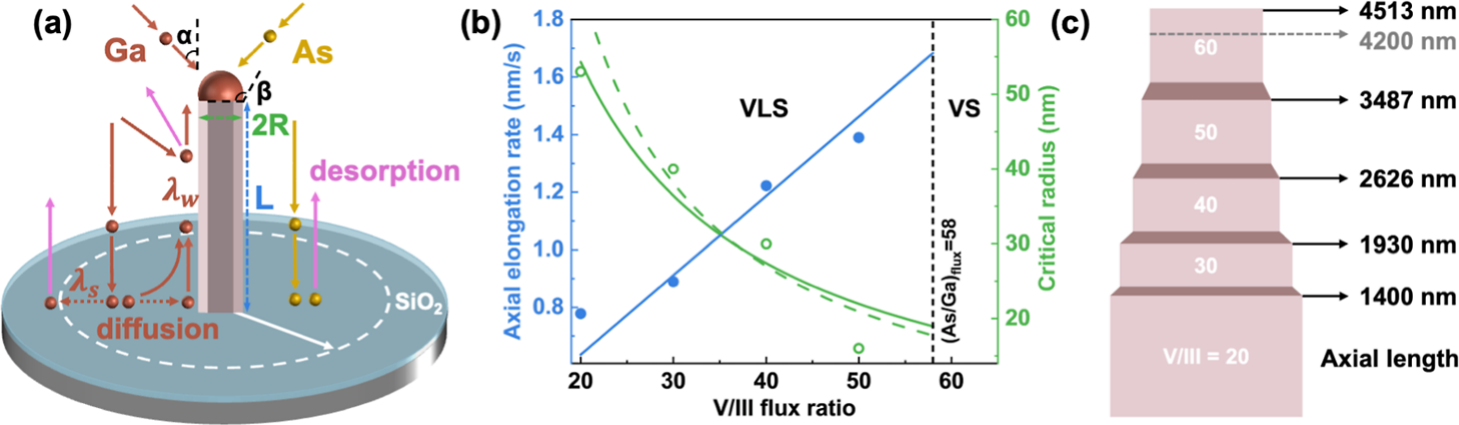
Growth model and analysis of elongation and radius evolution in GaAs nanowires. (a) Schematic illustration of the VLS growth model. α denotes the impingement angle of the Ga flux, and β is the contact angle between the Ga droplet and nanowire crystal. The nanowire radius *R* is determined by the Ga influx rate (χ_Ga_ν_Ga_), the axial elongation rate (d*L*/d*t*), and the diffusion of Ga adatoms from both the sidewall and the substrate surface. (b) Linear fit of the axial elongation rate as a function of the V/III flux ratio (solid blue line) and reciprocal fit of the measured nanowire radius (solid green curve), shown alongside the predicted variation in critical radius from eq 3 (dashed green curve). Average nanowire lengths and radii are plotted as blue dots and green open circles, respectively. (c) Axial length estimation of GaAs nanowires grown under a gradual increase in the V/III flux ratio. The actual growth is observed to terminate at a length of approximately 4200 nm (corresponding to a V/III ratio of 60), as indicated by the gray dashed arrow.

From eq 1, it is clear that the NW radius is dependent on two sets of parameters: (i) the axial elongation rate (d*L*/d*t*) relative to the Ga supply from the incoming flux (χ_Ga_ν_Ga_) and (ii) the effective diffusion length of Ga adatoms (λ), either along the NW sidewall or from the adjacent substrate surface. These parameters can be determined experimentally in our samples. Specifically, we observed that GaAs NW reference sample C1, grown under a V/III flux ratio of 20, reached an average length of ∼1.4 μm after 30 min of growth, corresponding to an axial elongation rate d*L*/d*t* = 0.78 nm/s. Based on a fixed Ga planar growth rate ν_Ga_ cos α = 0.085 nm/s, which yields ν_Ga_ = 0.12 nm/s at an incidence angle α = 45°, as shown in Figure 2a. Using an average contact angle of β = 135°, the impingement rate of the Ga flux is calculated as χ_Ga_ν_Ga_ = 0.24 nm/s. From these calculations, we find that d*L*/d*t* > χ_Ga_ν_Ga_, indicating that the Ga supply from only the direct flux may be insufficient to sustain the Ga droplet due to the faster crystallization rate. Therefore, droplet stability becomes increasingly dependent on Ga adatom diffusion. This introduces a self-equilibration effect within the VLS growth method, which drives the NW radius to converge toward a critical value *R*_c_.^24,25^ Specifically, during droplet-catalyzed GaAs NW growth under As-rich conditions, an equilibrium droplet size is established at each given V/III flux ratio. When the axial elongation rate exceeds the Ga supply rate from the vapor phase into the droplet, the droplet is further stabilized by diffusion of the adatom from the NW sidewalls. This convergence toward this critical radius *R*_c_ is described by eq 2, derived from eq 1 under the assumption that *p* = 1:

2 $$R_{c}=\frac{2\nu_{Ga}\lambda \sin\alpha }{\pi \left(\frac{dL}{dt}-\chi_{Ga}\nu_{Ga}\right)}$$

Experimentally, the average diameter of sample C1 grown under a V/III flux ratio of 20 was measured to be 106 nm, corresponding to a critical radius of *R*_c_ = 53 nm. Based on this value, the Ga diffusivity-related term 2ν_Ga_λ sin α is calculated to be 94 nm^2^/s, from which the effective diffusion length of Ga adatoms on the NW sidewall is deduced to be λ = 553 nm. It should be noted that this effective diffusion length is a simplified estimate that does not account for the initial growth stage nor for the contribution of Ga adatoms collected from the substrate surface.^37^ Using this diffusion length, along with a measured NW elongation length of ∼2.2 μm under a V/III flux ratio of 40 for sample A1 (corresponding to d*L*/d*t* = 1.22 nm/s), we further estimate the critical radius under a flux ratio of 40 to be *R*_c_ = 29 nm. This value agrees well with the experimentally measured radius of the diameter-reduced NW segment, which is ∼30 nm.

Three additional samples were grown to investigate the effects of droplet size and elongation-stage V/III flux ratio on the NW diameter with: (i) a higher droplet shrinkage ratio of 120 (sample A2), (ii) an extended droplet shrinkage duration of 7.5 min (sample A3), and (iii) an increased V/III flux ratio of 50 during the reduced-diameter elongation stage (sample A4), as summarized in Table 1. We observe a reduced radius of ∼16 nm in sample A4, and the axial elongation of the thin NW segment reaches ∼834 nm after 10 min growth, giving an elongation rate of d*L*/d*t* = 1.39 nm/s. In contrast, the radii of samples A2 and A3, both designed to modify the droplet shrinkage process, are similar to that of A1 at ∼28 and ∼25 nm, respectively, as shown in Figure S2 of the Supporting Information. This indicates that although a reduced droplet volume promotes the initiation of small-diameter NW growth, it is not the primary factor determining the final NW diameter. Under a higher V/III flux ratio of 40 (samples A2 and A3) or 50 (sample A4) during elongation, compared to the ratio of 20 used for the GaAs stem, the shrunken Ga droplet further inflates or shrinks until it stabilizes at an equilibrium size corresponding to the given flux ratio, producing a reduced NW diameter.

Since the Ga influx remains constant throughout the GaAs NW growth, the axial elongation rate can be considered simply proportional to the As influx and thus to the V/III flux ratio.^25,38^ Based on the measured axial elongation rates at V/III flux ratios of 20, 30 (sample C2, ∼3.2 μm after 60 min of growth, corresponding to an elongation rate of 0.89 nm/s), 40, and 50, a linear fit was performed to determine the relationship between the elongation rate and flux ratio. The fitted function is indicated by the solid blue line in Figure 2b, while the blue circles represent the experimentally measured elongation rates. This fitted linear relationship enables quantitative analysis of the critical radius *R*_c_ as a function of the V/III flux ratio. Using the measured NW radius at flux ratios of 20, 30 (∼40 nm), 40, and 50, we compute a corrected average effective Ga adatom diffusion length, λ, of 474 nm. We notice a decrease in the average λ compared to λ = 553 nm at a V/III flux ratio of 20, due to reduced Ga diffusivity under As-rich conditions required to maintain stoichiometric III–V growth.^39,40^ Since the Ga supply is fixed, we can substitute the parameters in eq 2 with actual values, yielding eq 3:

3 $$R_{c}=\frac{80.38}{\pi \left(0.02757x-0.15605\right)},\left(6\leq x\leq 58\right)$$

where *x* is the V/III flux ratio. This expression is valid for V/III flux ratios in the range of 6 to 58. Below *x* = 6, the Ga droplet is expected to continuously expand due to an excessive Ga supply rate relative to the Ga consumption rate by crystallization (χ_Ga_ν_Ga_ > d*L*/d*t*), leading to radial expansion of the NW.^26^ The upper limit of *x* = 58 corresponds to the point at which the effective As flux equals that of the Ga flux at the NW apex, resulting in a transition from droplet-catalyzed VLS growth to facet-driven VS growth. This transition was previously reported by Rudolph et al. who observed it when the As/Ga growth rate ratio exceeded 10.84, corresponding to an As growth rate of 2.71 Å/s and a Ga growth rate of 0.25 Å/s.^41^ In the current study, under a fixed Ga growth rate of 0.85 Å/s, an As growth rate of 9.21 Å/s corresponding to a V/III flux ratio of 58 is predicted to trigger the same transition to droplet-free VS growth. The experimentally measured NW radii (green open circles) are fitted by using a reciprocal function (solid green curve) and compared with the predicted variation in critical radius from eq 3 (dashed green curve), as shown in Figure 2b. This model reproduces the overall trend observed in the measured data, indicating that the critical radius *R*_c_ decreases with an increasing V/III flux ratio. Nevertheless, a systematic offset between the predicted and measured values is observed. This deviation likely arises from the instantaneous diameter variations during growth commonly observed in III–V NWs, which are not accounted for in the model.^42,43^ Despite this limitation, the model effectively captures the general radial evolution, confirming that the V/III flux ratio plays a key role in controlling the NW diameter under the applied growth conditions.

For the gradual growth method, assuming that sample B1 grows via the VLS growth method, the expected NW length at each growth period (as shown in Figure 2c) can be calculated based on its growth profile as presented in Figure 1b and c. Compared with the measured NW length of ∼4.2 μm, it can be inferred that axial growth terminates before the completion of the 95 min growth period. This is attributed to the progressive depletion of the Ga droplet under increasingly As-rich conditions, which begins when the V/III flux ratio reaches the VLS-to-VS transition point described above. As shown in Figure 2b, we predict that this transition occurs once the V/III flux ratio exceeds 58, which is in good agreement with the observed cessation of elongation at around a V/III ratio of 60 in Figure 2c. Beyond this point, GaAs NW growth proceeds via the facet-driven VS growth method, as shown in Figure S1e of the Supporting Information.

It is noteworthy that the observed radius of sample B1 does not strictly follow the predicted critical radius relationship shown in Figure 2b. This deviation arises because this relationship was developed for NWs grown under equilibrium V/III flux ratios with a stationary Ga droplet size, as in the case of sample A1, where a deliberate droplet shrinkage step was applied to establish a smaller droplet volume before changing the flux ratio for thin NW elongation. In contrast, sample B1 was grown under a continuously increasing As flux without interruption. Due to the absence of droplet modulation between each 8 min growth interval, the Ga droplet volume does not remain constant, gradually shrinking and requiring volume equilibration in the period after each 2 min increment of the V/III flux ratio. Consequently, the segments of sample B1 grown within the VLS growth regime tend to exhibit a tapered morphology with a slight reduction in diameter during each growth interval.

Following the growth of reduced-diameter GaAs NWs by abruptly increasing the V/III flux ratio, Ge shells were epitaxially grown around the GaAs cores (sample A1) to investigate the integration of the III–V and group-IV materials. The Ge shell exhibits an uneven sidewall morphology with a sawtooth-like pattern near the tip region. Statistical analyses of both core and core–shell NW diameters are presented in Figure 3. A significant diameter reduction is observed in the GaAs cores, with the lower region measuring 111 ± 16 nm and the upper region being reduced to 60 ± 9 nm. Based on the difference in radii between the GaAs cores and the GaAs/Ge core–shell NWs, the Ge shell thickness is estimated to be 62.5 nm in the lower region and 65 nm in the upper region of the NW.

**Figure 3:**
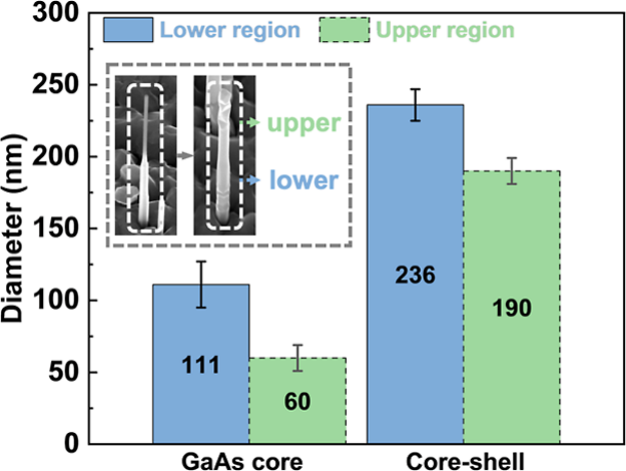
Statistical analysis for GaAs nanowires and GaAs/Ge core–shell nanowires. Diameter statistics of reduced-diameter GaAs nanowires and GaAs/Ge core–shell nanowires at the lower and upper NW regions. The inset panel shows SEM images of the nanowires, highlighted with dashed boxes. The error bars give the standard deviations for the statistics. Note that the SEM images were taken from a 30° tilted sample stage, and the statistical data were obtained from a minimum of 20 nanowires in each sample set.

To investigate the morphology of the Ge shell, high-magnification TEM measurements were performed along the ⟨110⟩ crystallographic direction. As shown in Figure 4a and b, stacking faults and twin defects are observed near the lower and upper regions of the Ge shell, accompanied by microfacets along the growth axis. These features are consistent with previously reported observations in GaAs/Ge core–shell NWs with a uniform-diameter GaAs core.^32^ Defects such as rotational twins can introduce local variations in formation energy, disrupt atomic continuity, and alter surface energies at twin boundaries.^44^ These energetic imbalances can modify facet growth rates, leading to the development of new sidewall facets or modifications in their inclination and morphology.^45^

**Figure 4:**
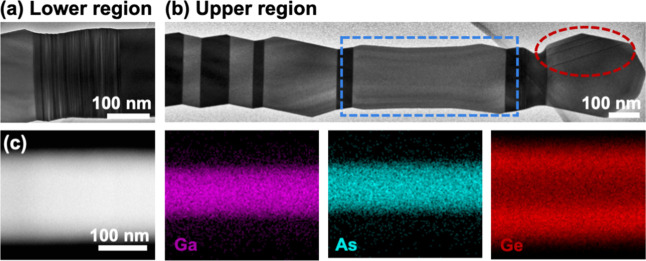
Crystal defect analyses and elemental distribution of the GaAs/Ge core–shell nanowire. (a,b) High-magnification TEM images of the lower and upper regions of the nanowire. Noticeable stacking faults are observed in the lower region of the Ge shell in (a). Faceting and twin defects are seen in the upper region in (b). In (b), planar defects on {111} planes at 70.5° with respect to the growth plane are clearly visible, appearing as a series of parallel dark lines within the red dashed circle. (c) EDX mapping of the reduced diameter region in the blue dashed box in (b), showing a uniform distribution of elements: Ga (magenta), As (cyan), and Ge (red).

Given the epitaxial nature of core–shell NWs, crystal defects in Ge shells are typically inherited from the III–V core,^46^ particularly during rapid changes of the catalyst droplet volume that disrupt the lattice continuity. High-resolution TEM analysis in Figure S3 of the Supporting Information reveals periodic Type-I1 stacking faults and a localized wurtzite segment embedded within the zinc blende GaAs core (sample A1). These crystal defects are likely to be transferred from the defective GaAs core to the subsequently epitaxially grown Ge shell. Prior studies have shown that crystal imperfections in the Ge shell, including twinning and surface faceting, can induce localized strain accumulation.^47^ This is supported by our Raman spectroscopy measurements presented in Figure S6 of the Supporting Information, which reveal stress-related shifts in the Ge shell. Such strain can influence the band structure by altering the conduction and valence band edges.^47^ In addition, stacking faults originating from polytypic transitions can form atomically abrupt interfaces that affect the optoelectronic properties of the heterostructure. These interfaces may behave as a superlattice, modifying Bloch wave functions due to the differing crystal symmetries and band structures across adjacent domains.^48^ Additionally, planar defects on {111} planes at 70.5° with respect to the growth are observed at the NW tip, highlighted by the red dashed ellipse in Figure 4b. These planar defects are induced by the transition between VLS and VS growth models during Ge shell deposition.^48^ When the residual Ga catalyst droplet is depleted toward the end of shell growth, two concurrent nucleation processes can occur within the unconsumed Ga droplet: one driven by As retained in the droplet, promoting GaAs crystallization, and the other by Ge vapor, driving Ge crystallization. This simultaneous crystallization of GaAs and Ge forms planar defects at the NW apex.

EDX mapping of the reduced-diameter region, marked by the blue dashed box in Figure 4b, is shown in Figure 4c. Elemental maps of Ga (magenta), As (cyan), and Ge (red) confirm a uniform distribution along the axis of the core–shell heterostructure. Similar crystal structure analysis and side-view EDX mapping are presented in Figure S4 of the Supporting Information, corresponding to a GaAs/Ge core–shell NW with a reduction of the GaAs diameter. Interestingly, the Ge shell does not conform to the diameter reduction of the GaAs core. Instead, it maintains a consistent thickness along the NW growth direction. This observation is supported by statistical data in Figure 3, which show similar Ge shell thicknesses in the lower (65 nm) and upper (62.5 nm) regions of the NW. This uniformity in shell thickness is attributed to the underlying growth dynamics. During VS growth in MBE, there are two primary mechanisms determining the Ge collection: (i) direct impingement of Ge atoms onto the NW sidewall and (ii) surface diffusion of Ge adatoms either along the sidewall or from the nearby substrate following initial impingement. To quantify the surface diffusion length of Ge adatoms, we apply a previously reported diffusion model under the following assumptions: (i) diffusion occurs in a steady-state manner from the substrate to the NW and along the NW sidewall; (ii) the spacing between adjacent wires does not affect substrate surface diffusion; and (iii) the Ge shell can be treated as Ge growing on a Ge surface over the growth period. Under these assumptions, the diffusion lengths of Ge adatoms on the substrate surface (λ_s_^Ge^) and on the NW sidewalls (λ_w_^Ge^) can be described by eqs 4 and 5, respectively.^49,50^

4 $$\lambda_{s}^{Ge}=\frac{1}{\sqrt{2\pi N_{w}+2\pi N_{i,0}{\;e}^{2F_{s}\left(\frac{T_{0}}{T}-1\right)}+\lambda_{0}^{-2}\;e^{-2G_{s}\left(\frac{T_{0}}{T}-1\right)}}}$$

5 $$\lambda_{w}^{Ge}=\lambda_{0}\;e^{G_{s}\left(\frac{T_{0}}{T}-1\right)}$$

with the NW density *N*_w_, the island density on the substrate surface *N*_i,0_ at temperature *T*_0_, and the pristine diffusion length of Ge adatoms λ_0_ at temperature *T*, the parameters *G*_s_ and *F*_s_ are defined by eqs 6 and 7, respectively.

6 $$G_{s}=\frac{\left(E_{des}-E_{dif}\right)}{2k_{B}T_{0}}$$

7 $$F_{s}=\frac{\left(3\Lambda_{s}+2E_{dif}\right)}{2k_{B}T_{0}}$$

where *E*_des_ and *E*_dif_ are the activation energies for desorption and diffusion, respectively, *k*_B_ is the Boltzmann constant, and Λ_s_ is the condensation heat of surface atoms.

Several parameters at a growth temperature of *T*_0_ = 430 °C have been reported by Schmidtbauer et al., including: λ_0_ = 126 nm, *G*_s_ = 4.8, and *F*_s_ = 11.6.^49^ Additional parameters can be derived from the present work: *T* = 400 °C, *N*_w_ = 1.02 × 10^–12^ m^2^, and *N*_i,0_ = 5 × 10^–13^ m^2^. Using these values and applying eqs 4 and 5, the diffusion lengths of Ge adatoms are estimated as follows: λ_s_^Ge^ = 90 nm on the substrate surface and λ_w_^Ge^ = 90 nm on the NW sidewall. Rezvani et al. further reported that the diffusion length of Ge adatoms is also influenced by the deposition flux,^51^ and surface-related barriers, for example, defects, can affect the actual diffusivity of Ge. Therefore, the actual diffusion lengths may deviate from the calculated values. The calculated diffusion lengths of Ge, whether on the adjacent substrate or along the NW sidewalls, are insufficient to support significant diffusion-driven shell growth, compared to the total NW length. As a result, Ge shell growth under the VS method is primarily governed by the direct impingement of the growth species. During this process, Ge atoms are expected to crystallize close to the locations where they reach the NW sidewall, leading to a uniform shell thickness along the NW axis that does not mirror the diameter reduction of the GaAs core template.

Cross-sectional analysis of interface quality and elemental composition was carried out on sections taken from the lower part of resin-embedded GaAs/Ge core–shell NWs by using an ultramicrotome. Figure 5a and b show high-magnification annular dark-field scanning transmission electron microscopy (ADF-STEM) images of a typical GaAs/Ge core–shell NW cross-section. We note that the hexagonal GaAs core is bounded by six low-energy {110} facets with respect to the ⟨111⟩ growth direction, whereas the radially grown Ge shell on GaAs core adopts six high-index {112} facets to minimize the surface energy, consistent with observations reported for group-IV shells.^52–54^ This facet reconstruction in the Ge shell induces a 47° core–shell rotation during growth, as shown in Figure 5a, and contributes to the strain relaxation related to the crystal defects observed in Figure 4.^55^ In addition, the GaAs core is slightly off-center within the Ge shell, which is attributed to sidewall faceting, which causes a variation in shell thickness. In Figure 5c, an atomic-resolution ADF-STEM image reveals a smooth GaAs/Ge interface with no observable misfit dislocations, confirming a high core–shell interface quality. To assess elemental distributions, EDX mapping was performed on the core–shell cross section. Figure 5d displays the elemental maps of Ga (magenta), As (cyan), and Ge (red). The elements are uniformly distributed, although slight intermixing is observed at the core–shell interface, as indicated by the EDX cross-sectional line scan in Figure S5 of the Supporting Information. Additional structural and optical characterization of the GaAs/Ge core–shell NWs using XRD, Raman spectroscopy, and PL measurements is presented in Figure S6 of the Supporting Information.

**Figure 5:**
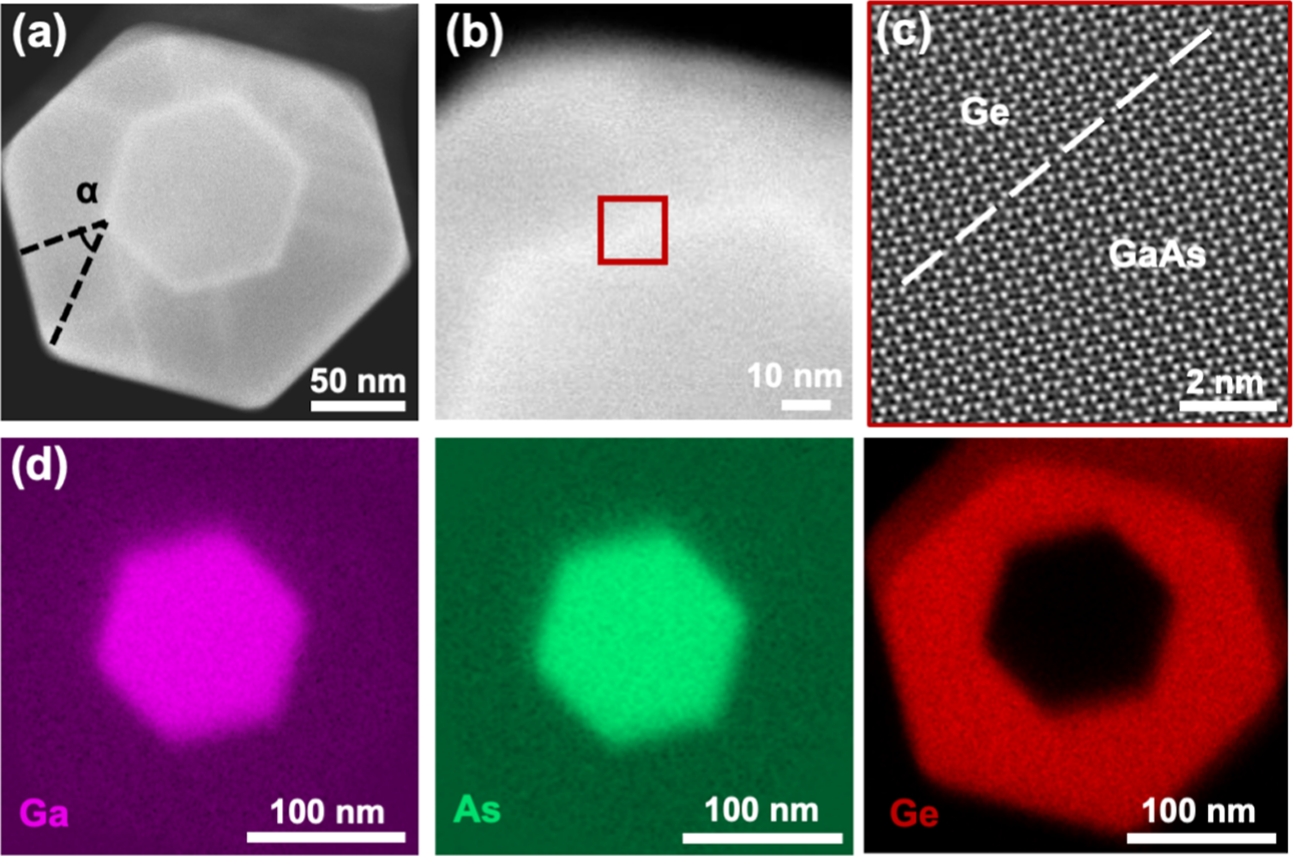
Core–shell interface images and compositional analysis of the GaAs/Ge core–shell nanowire cross section. (a,b) ADF-STEM images of the cross-section of a GaAs/Ge core–shell nanowire. The exterior Ge shell is inclined by an angle (α) of 47° relative to the internal GaAs core. (c) High-resolution ADF-STEM image, taken from the red inset box in (b). The white dashed line indicates the interface between the GaAs core and the Ge shell. (d) EDX mapping of the cross-section from (a), showing uniform distributions of elements: Ga (magenta), As (cyan), and Ge (red).

## 4 Conclusions

To summarize, MBE growth of reduced-diameter GaAs NWs and their subsequent integration with Ge shells have been demonstrated. The diameter reduction is driven by two primary factors: first, rapid shrinkage of the Ga droplet volume under elevated V/III flux conditions; and second, a smaller critical radius associated with an increased flux ratio. We present a quantitative relationship that links NW radius to changes in the V/III flux ratio, enabling the prediction of diameter evolution under different flux ratios. In contrast, under a gradual increase in the V/III flux ratio, pronounced radial expansion is observed at the NW apex. Under increasingly As-rich conditions, the Ga influx and surface diffusion become insufficient to sustain the droplet, leading to its depletion. This depletion marks the termination of axial growth and a transition from VLS-dominated growth to VS growth after the V/III flux ratio exceeds 58. The subsequent heteroepitaxial growth of Ge shells results in a uniform shell thickness, irrespective of the reduced GaAs core diameter. Structural characterization reveals the presence of crystal defects along the axial direction of the Ge shell, associated with microfaceting at these sites. Nevertheless, the core–shell interface remains coherent, exhibiting no misfit dislocations and minimal strain in the III–V/IV heterostructure. High-resolution ADF-STEM imaging reveals a 47° rotation of the hexagonal Ge shell facets relative to that of the GaAs core. These findings provide a method for diameter control in self-catalyzed III–V NWs and offer insight into the NW growth mechanisms governed by the V/III flux ratio.

## Supplementary Material



## Notes

SEM images of GaAs nanowires grown using the two V/III modulation methods; crystal structure analysis of the GaAs/Ge core–shell nanowire; EDX line scan of the GaAs/Ge core–shell nanowire; and strain and PL analysis of the nanowires (PDF)
