# Supplementary material for: As-Flux-Induced Diameter Control in GaAs Nanowires
Source: J Phys Chem C Nanomater Interfaces. 2025 Sep 23;129(39):17607–15. doi: 10.1021/acs.jpcc.5c03887 (PMC12498496; doi:10.1021/acs.jpcc.5c03887)
Supplement: Supplementary file 1 [file jp5c03887_si_001.pdf]

# Supporting Information

# As-flux-induced Diameter Control in GaAs Nanowires

*Ziyue Yin[1,▿], Haotian Zeng[1,▿,*], Giorgos Boras[1,*], Raghavendra R Juluri[2], Huiwen Deng[1,*], Hui Jia[1], Chong Chen[1], Stephen Church[3], Anton Velychko[4], Fahad Alghamdi[1,5], Jae-Seong Park[1], Mingchu Tang[1], David Mowbray[4], Patrick Parkinson[3], Ana M Sanchez[2] and Huiyun Liu[1]*

[1] Department of Electronic and Electrical Engineering, University College London, London WC1E 7JE, United Kingdom

[2] Department of Physics, University of Warwick, Coventry CV4 7AL, United Kingdom

[3] Department of Physics and Astronomy and the Photon Science Institute, The University of Manchester, M13 9PL, United Kingdom

[4] School of Mathematical and Physical Sciences, The University of Sheffield, Sheffield S3 7RH, United Kingdom

[5] King Abdulaziz City for Science and Technology, Riyadh 11442, Kingdom of Saudi Arabia

[▿] These authors contributed equally.

[*] Author to whom any correspondence should be addressed.

Email: haotian.zeng@ucl.ac.uk, g.boras@ucl.ac.uk, huiwen.deng@ucl.ac.uk

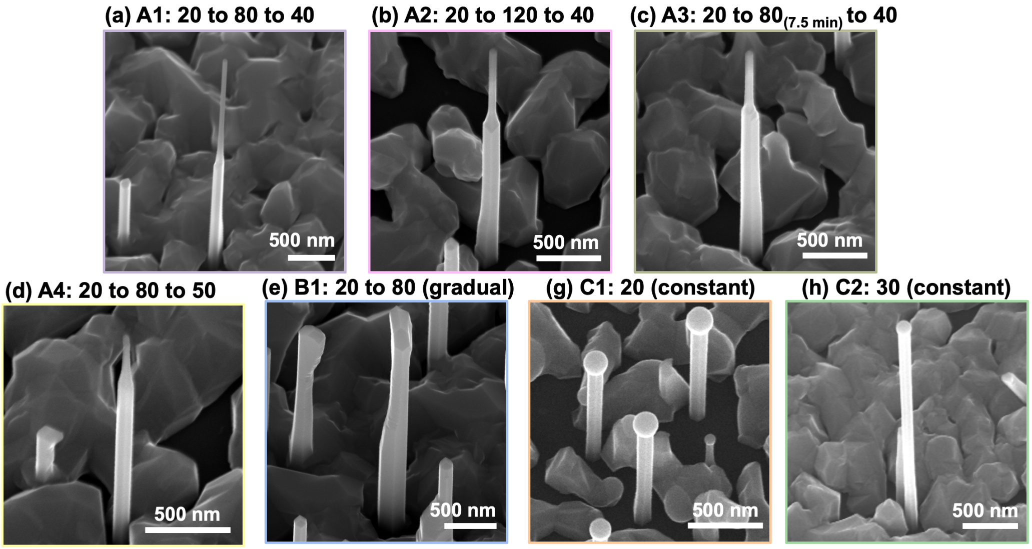


**Figure S1. SEM characterization of GaAs nanowires.** *(a-d) SEM images of samples A1-A4. (e) SEM images of samples B1. (g-h) SEM images of samples C1 and C2. All SEM images were taken at a 30° tilt. Detailed sample information is summarized in Table 1 of the main text.*

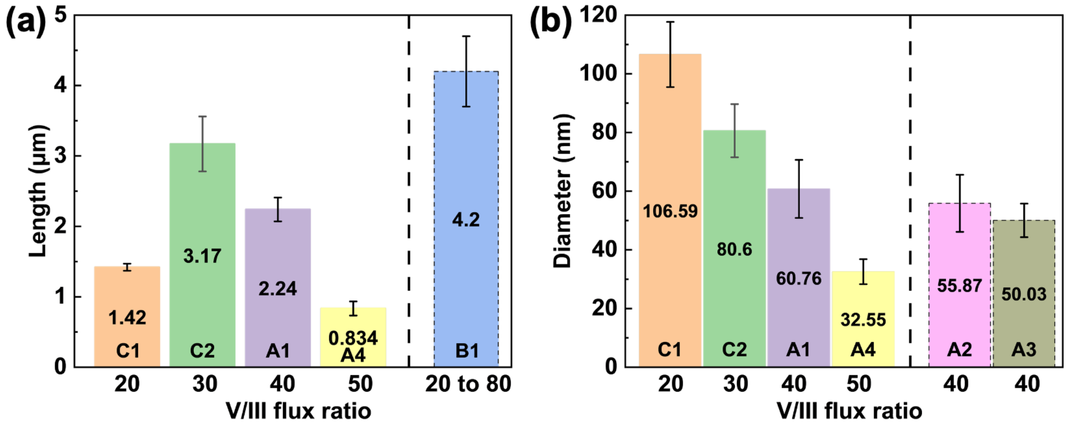


**Figure S2. Statistical analysis of nanowire length and diameter.** *(a) Column bar chart showing nanowire length statistics. For samples A1 and A4 with diameter reduction designs, the lengths were measured from their reduced-diameter segments grown under elevated V/III flux ratios. (b) Column bar chart showing nanowire diameter statistics. Sample identifiers are indicated below each column.*

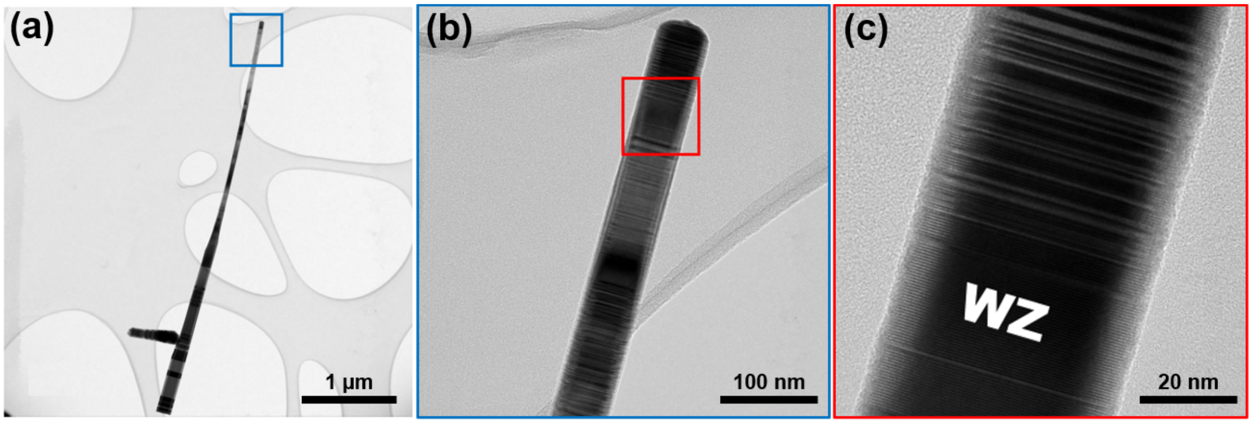


**Figure S3. Crystal structure analysis of GaAs nanowire (sample A1).** *(a) Low-magnification TEM image of a GaAs nanowire exhibiting pronounced diameter reduction toward the tip. (b) High-magnification TEM image taken from the apex region (indicated by the blue box in panel a). (c) Atomic-resolution TEM image of the red box region in panel (b), revealing periodic contrast bands consistent with type-I1 stacking faults and phase transitions. The alternating stacking sequences correspond to zinc blende and wurtzite-like polytypic structures.*

In III-V/IV core-shell NWs, crystal structure transfer across the core-shell interface typically results in the shell following both the atomic stacking sequence and any pre-existing crystallographic defects from the core.[1] As shown in Figure 4 of the main text, the Ge shell exhibits regions of periodic contrast in both the lower and upper segments, which are attributed to crystal defects such as twin boundaries and stacking faults inherited from the GaAs core. To investigate their origin, we examined the bare GaAs NW core (sample A1) using transmission electron microscopy (TEM). Figure S3a reveals a pronounced diameter reduction near the tip, and high-magnification imaging of this region in Figure S3b shows repeated transitions between zinc blende (ZB) and wurtzite (WZ) stacking. Atomic-resolution TEM in Figure S3c further reveals a relatively long, uninterrupted WZ segment, indicating a local phase transformation from ZB to

WZ. Such transitions are due to twinning-induced polytype formation rather than isolated stacking faults. In contrast, the upper region of the NW exhibits periodic ZB-WZ-like transitions, characteristic of Type-I1 stacking faults.[1,2] Similar long-range polytypic segments with ~2 nm thickness for each structure are reported in VLS-grown GaAs NWs.[3]

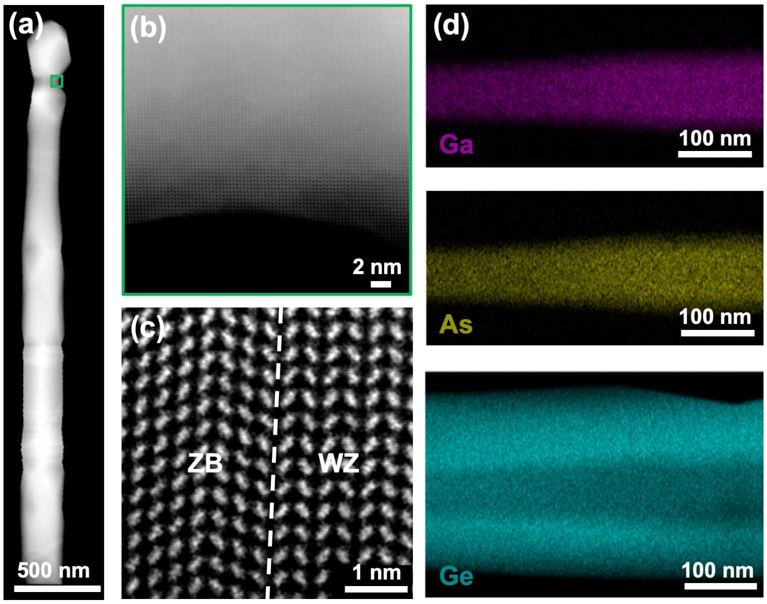


**Figure S4. Crystal structure analysis of GaAs/Ge core-shell nanowires.** *(a) Low-magnification TEM image of a GaAs/Ge core-shell nanowire, with the green inset highlighting the region examined in (b). (b) High-magnification TEM image of the selected area, revealing wurtzite segments up to 88 nm in length. (c) Detailed characterization of the Ge shell's crystal structure, showing the boundary between zinc blende and wurtzite phases and the presence of a twin defect in the ZB segment, as indicated by the red arrow. (d) Side-view EDX mapping at the diameter reduction region of the GaAs core, showing elemental distributions of Ga (magenta), As (yellow), and Ge (cyan). A slight variation in Ge shell thickness is noted near the diameter reduction region.*

It has been reported that the Ga droplet contact angle, which determines the crystal phase of the new crystallization layer, could dynamically change during Ga catalyst droplet manipulation.[4] To

understand the crystal structure of the Ge shell, TEM analysis were conducted. As shown in Figures S4a and S4b, TEM images of a GaAs/Ge core-shell NW reveal the sidewall morphology, while the higher-magnification image in Figure S4c captures a transition from the ZB phase to the WZ phase. EDX mapping was also performed near the reduced diameter region of the GaAs core to study the formation of the Ge shell, as shown in Figure S4d. The elements are represented by the following colors: Ga (magenta), As (yellow), and Ge (cyan). The observation of a consistent Ge shell thickness along the NW axis is elaborated in the main paper.

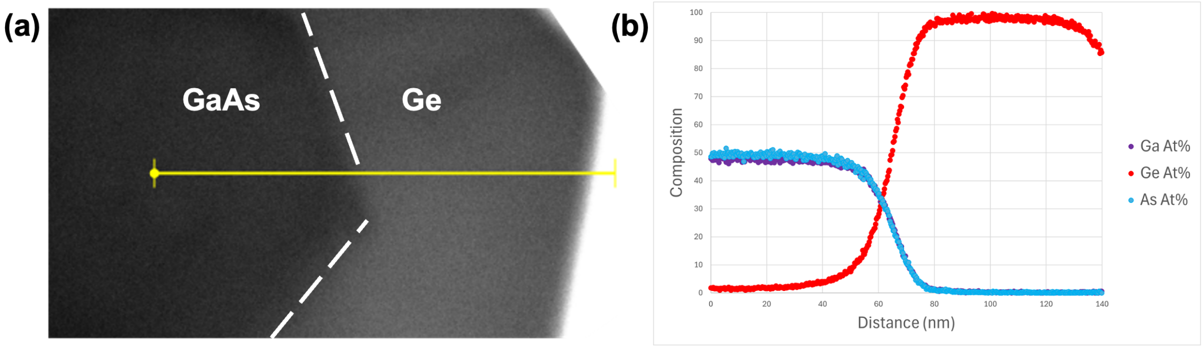


**Figure S5. EDX line scan of the cross-section of a GaAs/Ge core-shell nanowire.** *(a) ADF-STEM image of the GaAs/Ge core-shell cross-section, where the GaAs core appears as a darker region. (b) A plot of the elemental compositions. The composition analysis data was obtained from an EDX line scan along the yellow line in the GaAs/Ge core-shell nanowire in (a).*

As shown in Figure S5a, the GaAs core and Ge shell exhibit a relative rotation, this is discussed in the main paper. Figure S5b shows an EDX line scan of the elemental distribution across the core-shell interface in the reduced diameter region. The results indicate the incorporation of Ge atoms into the GaAs core near the interface region, and small traces of Ga and As atoms are found in the Ge shells.

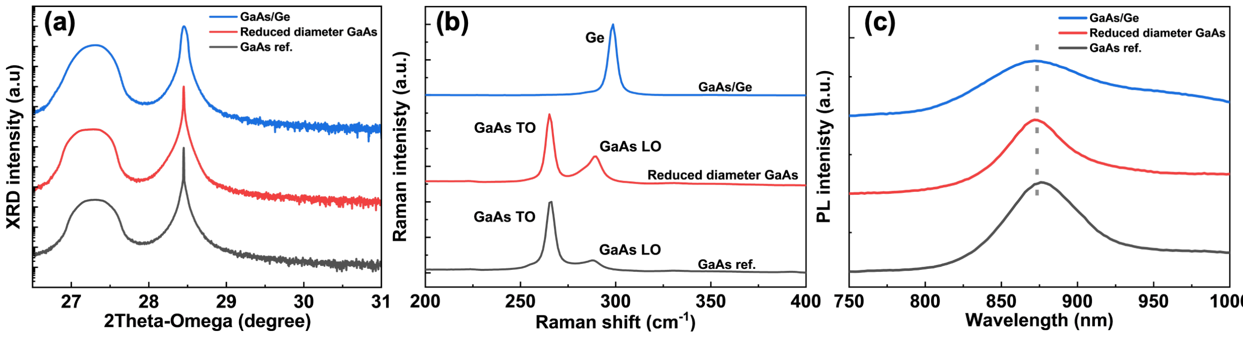


**Figure S6. Strain and PL analysis of GaAs nanowires and GaAs/Ge core-shell nanowires.** *(a) XRD spectra of GaAs/Ge core-shell nanowires (blue), reduced diameter GaAs nanowires (red), and GaAs reference nanowires (grey) grown on Si (111) substrates. (b) Raman spectra of the three nanowire structures showing the transverse optical and longitudinal optical phonon modes. (c) Room-temperature PL spectra with the dotted grey line indicating the peak emission wavelength for the nanowire structures. Note that the peak intensities in the XRD plots are normalized to their respective maximum values.*

XRD analysis and Raman spectroscopy were performed to investigate the strain distribution at the GaAs/Ge core-shell interface. Figure S6a shows XRD scans for GaAs NWs and GaAs/Ge core-shell NWs. Two noticeable peaks are presented in the XRD scans, which are attributed to the Si (111) substrate (at 28.45 degrees, with a high intensity and small FWHM) and the NWs (from 26.66 to 27.87 degrees, with a low intensity and large FWHM). For the reduced diameter GaAs NW (red), a slight broadening of the NW peak is observed, this can be attributed to the mixture of crystal phases in the reduced diameter region.[5,6] Compared to the GaAs NWs, the presence of the Ge shell (blue) produces an even broader peak. Given the similar lattice constants of GaAs and Ge, this peak is believed to result from the combined contributions of both the GaAs core and the Ge shell. Raman spectra are shown in Figure S6b. For the GaAs/Ge core-shell NWs (blue), a sharp and narrow peak due to the Ge optical phonon is present at 297.5 $cm^{-1}$. Because of the crystal

transfer within the core-shell heterostructure,[7] the Ge shell will adopt the atomic arrangement of the GaAs core to give a mixture of crystal phases. The phonon frequency (297.5cm$^{-1}$) is in good agreement with a reported value of 297 cm$^{-1}$ for Ge NWs with polytypic crystal structures.[8] In comparison to fully relaxed Ge (phonon frequency 292.0 cm$^{-1}$),[9] the higher frequency observed in Figure S6b may also result from the presence of compressive strain in the Ge shells. This accumulated strain may be relieved via the formation of micro-facets as seen in Figure 4b of the main paper. No distinct peaks are observed from the GaAs NW in the core-shell structure, indicating that the thick Ge shell provides complete coverage of the core and that the laser and scattered light is strongly attenuated by the shell.[10,11] A dominant TO phonon at 265.1 cm$^{-1}$ and the longitudinal optical (LO) phonon at 289.7 cm$^{-1}$ are observed for the reduced diameter GaAs core (red), which are consistent with reported frequencies of 30% wurtzite-phase GaAs NWs.[12] For the GaAs reference, the TO phonon appears at 266.3 cm$^{-1}$, with a weaker LO mode at 288.6 cm$^{-1}$. The TO phonon in the reduced diameter GaAs core exhibits a slight shift compared to the GaAs NW reference, which is attributed to internal strain induced by variations in lattice constants between different crystal structures. For the three NW structures, both internal and external strains are negligible, as only minor shifts in the phonon frequencies compared to fully relaxed structures are observed.

Room-temperature PL spectra of the GaAs NW reference, the reduced diameter GaAs NWs, and GaAs/Ge core-shell NWs are shown in Figure S6c. The GaAs emission of the three samples occurs close to 872 nm, as indicated by the dotted line. A reduction in intensity is observed for the sample with a Ge shell. This is expected, as the photon-generated carries may diffuse into the thick Ge shell which possess a narrower bandgap compared to that of GaAs. In addition, crystal defects

in the Ge shell may act as non-radiative recombination centers which leads to a weaker GaAs emission from GaAs/Ge core-shell NWs.

**References:**


(1) Conesa-Boj, S.; Hauge, H. I. T.; Verheijen, M. A.; Assali, S.; Li, A.; Bakkers, E. P. A. M.; Fontcuberta I Morral, A. Cracking the Si Shell Growth in Hexagonal GaP-Si Core–Shell Nanowires. *Nano Lett.* **2015**, *15* (5), 2974–2979. https://doi.org/10.1021/nl504813e.

(2) Furthmeier, S.; Dirnberger, F.; Hubmann, J.; Bauer, B.; Korn, T.; Schüller, C.; Zweck, J.; Reiger, E.; Bougeard, D. Long Exciton Lifetimes in Stacking-Fault-Free Wurtzite GaAs Nanowires. *Appl. Phys. Lett.* **2014**, *105* (22), 222109. https://doi.org/10.1063/1.4903482.

(3) Chen, Y.; Burgess, T.; An, X.; Mai, Y.-W.; Tan, H. H.; Zou, J.; Ringer, S. P.; Jagadish, C.; Liao, X. Effect of a High Density of Stacking Faults on the Young's Modulus of GaAs Nanowires. *Nano Lett.* **2016**, *16* (3), 1911–1916. https://doi.org/10.1021/acs.nanolett.5b05095.

(4) Panciera, F.; Baraissov, Z.; Patriarche, G.; Dubrovskii, V. G.; Glas, F.; Travers, L.; Mirsaidov, U.; Harmand, J.-C. Phase Selection in Self-Catalyzed GaAs Nanowires. *Nano Lett.* **2020**, *20* (3), 1669–1675. https://doi.org/10.1021/acs.nanolett.9b04808.

(5) Yeh, C.-Y.; Lu, Z. W.; Froyen, S.; Zunger, A. Zinc-Blende–Wurtzite Polytypism in Semiconductors. *Phys. Rev. B* **1992**, *46* (16), 10086–10097. https://doi.org/10.1103/PhysRevB.46.10086.

(6) Jahn, U.; Lähnemann, J.; Pfüller, C.; Brandt, O.; Breuer, S.; Jenichen, B.; Ramsteiner, M.; Geelhaar, L.; Riechert, H. Luminescence of GaAs Nanowires Consisting of Wurtzite and Zinc-Blende Segments. *Phys. Rev. B* **2012**, *85* (4), 045323. https://doi.org/10.1103/PhysRevB.85.045323.

(7) Algra, R. E.; Hocevar, M.; Verheijen, M. A.; Zardo, I.; Immink, G. G. W.; van Enckevort, W. J. P.; Abstreiter, G.; Kouwenhoven, L. P.; Vlieg, E.; Bakkers, E. P. A. M. Crystal Structure Transfer in Core/Shell Nanowires. *Nano Lett.* **2011**, *11* (4), 1690–1694. https://doi.org/10.1021/nl200208q.

(8) de Matteis, D.; De Luca, M.; Fadaly, E. M. T.; Verheijen, M. A.; López-Suárez, M.; Rurali, R.; Bakkers, E. P. A. M.; Zardo, I. Probing Lattice Dynamics and Electronic Resonances in Hexagonal Ge and SixGe1–x Alloys in Nanowires by Raman Spectroscopy. *ACS Nano* **2020**, *14* (6), 6845–6856. https://doi.org/10.1021/acsnano.0c00762.

(9) Zeng, H.; Yu, X.; Fonseka, H. A.; Gott, J. A.; Tang, M.; Zhang, Y.; Boras, G.; Xu, J.; Sanchez, A. M.; Liu, H. Hybrid III–V/IV Nanowires: High-Quality Ge Shell Epitaxy on GaAs Cores. *Nano Lett.* **2018**, *18* (10), 6397–6403. https://doi.org/10.1021/acs.nanolett.8b02760.

(10) Attolini, G.; Bosi, M.; Musayeva, N.; Pelosi, C.; Ferrari, C.; Arumainathan, S.; Timò, G. Homo and Hetero Epitaxy of Germanium Using Isobutylgermane. *Thin Solid Films* **2008**, *517* (1), 404–406. https://doi.org/10.1016/j.tsf.2008.08.137.

(11) Chang, Y.; Zhang, M.; Deng, C.; Men, C.; Chen, D.; Zhu, L.; Yu, W.; Wei, X.; Di, Z.; Wang, X. Fabrication of High Quality GaAs-on-Insulator via Ion-Cut of Epitaxial GaAs/Ge Heterostructure. *Appl. Surf. Sci.* **2015**, *346*, 46–49. https://doi.org/10.1016/j.apsusc.2015.03.198.

(12) Zardo, I.; Conesa-Boj, S.; Peiro, F.; Morante, J. R.; Arbiol, J.; Uccelli, E.; Abstreiter, G.; Fontcuberta I Morral, A. Raman Spectroscopy of Wurtzite and Zinc-Blende GaAs Nanowires: Polarization Dependence, Selection Rules, and Strain Effects. *Phys. Rev. B* **2009**, *80* (24), 245324. https://doi.org/10.1103/PhysRevB.80.245324.
